# Neurofilament Proteins as Biomarkers to Monitor Neurological Diseases and the Efficacy of Therapies

**DOI:** 10.3389/fnins.2021.689938

**Published:** 2021-09-27

**Authors:** Aidong Yuan, Ralph A. Nixon

**Affiliations:** ^1^Center for Dementia Research, Nathan Kline Institute, Orangeburg, NY, United States; ^2^Department of Psychiatry, NYU Neuroscience Institute, New York, NY, United States; ^3^Department of Cell Biology, New York University Grossman School of Medicine, (NYU), Neuroscience Institute, New York, NY, United States

**Keywords:** neurofilament, NFL, pNfH, biomarker, CSF, blood, neurodegeneration, neuronal injury

## Abstract

Biomarkers of neurodegeneration and neuronal injury have the potential to improve diagnostic accuracy, disease monitoring, prognosis, and measure treatment efficacy. Neurofilament proteins (NfPs) are well suited as biomarkers in these contexts because they are major neuron-specific components that maintain structural integrity and are sensitive to neurodegeneration and neuronal injury across a wide range of neurologic diseases. Low levels of NfPs are constantly released from neurons into the extracellular space and ultimately reach the cerebrospinal fluid (CSF) and blood under physiological conditions throughout normal brain development, maturation, and aging. NfP levels in CSF and blood rise above normal in response to neuronal injury and neurodegeneration independently of cause. NfPs in CSF measured by lumbar puncture are about 40-fold more concentrated than in blood in healthy individuals. New ultra-sensitive methods now allow minimally invasive measurement of these low levels of NfPs in serum or plasma to track disease onset and progression in neurological disorders or nervous system injury and assess responses to therapeutic interventions. Any of the five Nf subunits – neurofilament light chain (NfL), neurofilament medium chain (NfM), neurofilament heavy chain (NfH), alpha-internexin (INA) and peripherin (PRPH) may be altered in a given neuropathological condition. In familial and sporadic Alzheimer’s disease (AD), plasma NfL levels may rise as early as 22 years before clinical onset in familial AD and 10 years before sporadic AD. The major determinants of elevated levels of NfPs and degradation fragments in CSF and blood are the magnitude of damaged or degenerating axons of fiber tracks, the affected axon caliber sizes and the rate of release of NfP and fragments at different stages of a given neurological disease or condition directly or indirectly affecting central nervous system (CNS) and/or peripheral nervous system (PNS). NfPs are rapidly emerging as transformative blood biomarkers in neurology providing novel insights into a wide range of neurological diseases and advancing clinical trials. Here we summarize the current understanding of intracellular NfP physiology, pathophysiology and extracellular kinetics of NfPs in biofluids and review the value and limitations of NfPs and degradation fragments as biomarkers of neurodegeneration and neuronal injury.

## Introduction

It is widely accepted that the pathophysiology underlying many neurodegenerative disorders, such as Alzheimer’s disease (AD), originates many years prior to clinical symptoms. AD evolves through three stages – an early, preclinical stage with no detectable symptoms; a middle stage of mild cognitive impairment; and a late stage marked by symptoms of dementia. The lack of success in identifying treatments that cure AD or alter its progression has been attributed in part to the implementation of candidate treatments at a disease stage that is too advanced to blunt the disease triggering mechanism(s) or halt early progression before momentum builds to irreversible levels. There is a growing need for reliable non-invasive blood-based biomarkers for AD that can facilitate diagnosis, predict disease progression, and provide evidence of disease modification.

Neurofilament proteins (NfPs) appeared in the last few years as the most promising blood biomarkers of neuroaxonal integrity or damage. Nfs are classified as a type IV class of intermediate filaments (IFs) specific to neurons (Yuan et al., 2017). They are protein polymers measuring 10 nm in diameter and many micrometers in length. Together with microtubules (25 nm) and microfilaments (7 nm), they form the neuronal cytoskeleton. Much interest in the field has been recently focused on the detection of NfPs and degradation fragments released from neurons into blood as surrogate markers of neuronal damage in neuropathic states. The rationale for NfPs and fragments as biomarkers of neuronal damage is that they are not only responsive to neuronal injury but are also prominent components of abnormal intraneuronal aggregates in varied neurodegenerative diseases, including AD, dementia with Lewy bodies (DLB), Parkinson’s disease (PD), frontotemporal dementia (FTD), amyotrophic lateral sclerosis (ALS), Charcot-Marie-Tooth disease (CMT), multiple sclerosis (MS), giant axonal neuropathy (GAN) and toxic neuropathies. Although amyloid-beta and tau proteins are widely regarded as useful diagnostic biomarkers of AD, tau proteins increase only in specific neurodegenerative diseases such as AD and unaltered in other neurological diseases that are clearly neurodegenerative, such as tau-negative FTD caused by granulin or C9orf72 mutations (Foiani et al., 2018) where, by contrast, CSF and serum neurofilament light chain (NfL) fragment levels are more than 8 times higher in patients than in pre-symptomatic carriers or healthy controls (Meeter et al., 2016). Furthermore, in Huntington disease (HD), CSF NfL fragment levels correlate more strongly with disease progression than do CSF tau levels (Niemela et al., 2017). Moreover, studies using a stable isotope labeling method to investigate tau metabolism demonstrate that the production rate of tau positively correlates with the amount of amyloid plaques, suggesting that increased tau levels in AD could be due to elevated transcription, synthesis or secretion from neurons in response to amyloid-beta pathology rather than reflect actual neurodegeneration (Sato et al., 2018). Thus, as general neuronal integrity markers, NfPs and their fragments may be more sensitive to neurodegeneration than is tau.

In individuals with inherited forms of AD, levels of NfL fragments in blood may be altered 22 years before symptoms begin (Quiroz et al., 2020). NfL responds more sensitively to subclinical cognitive decline than amyloid-beta or tau (Bos et al., 2019; Kern et al., 2019; Merluzzi et al., 2019). Moreover, mean NfL fragment plasma levels increased 3.4 times faster in subjects who developed AD compared to those who remained dementia-free in a trajectory analysis of 4444 non-demented participants in the Rotterdam study at baseline and up to 14 years follow-up. In this review, we summarize the current understanding of NfPs and fragments as biomarkers in neurodegeneration and neurological injuries and draw attention to important unanswered questions.

## Properties of Neurofilaments Relevant to Their Use as Biomarkers

### The Physiological Basis of Neurofilament Proteins as Biomarkers of Neuronal Structural Integrity

**FIGURE 1:**
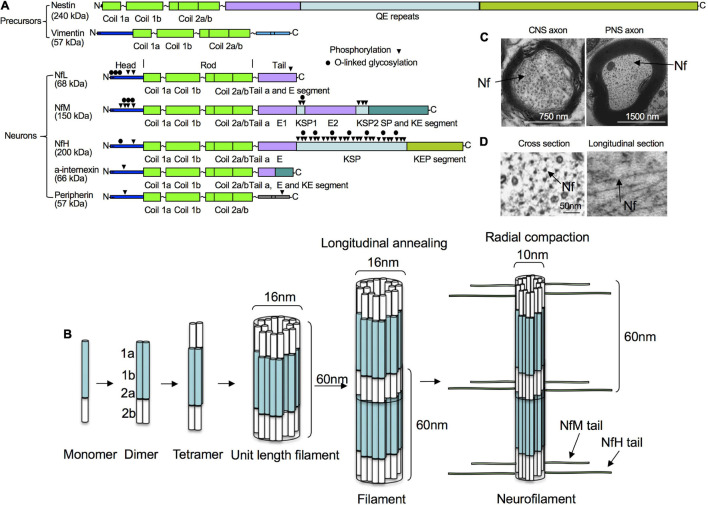
Structure, assembly and cytoarchitecture of Nfs. **(A)** Domain structure of Nfs in precursor and mature neurons. Precursor neurons contain nestin and vimentin while mature neurons have NfPs consisting of NfL, NfM, NfH, INA, and/or PRPH. All Nf subunits include a conserved alpha-helical rod domain, amino-terminal globular head regions and carboxy-terminal tail domains. Phosphorylation and O-linked glycosylation sites are shown. **(B)** Nf assembly. Nf monomers form coiled-coil heterodimers, then tetramers and unit-length filaments and gradual end-to-end annealing of which results in filament elongation to form mature Nfs with a diameter of about 10 nm after radial compaction. **(C)** Moderate number of Nfs in corpus callosum axons vs. large number of Nfs in sciatic axons in mice. **(D)** Ultrastructural representations of Nfs from mouse optic nerves in cross and longitudinal sections.

For a blood-based biomarker to reflect the structural integrity of neurons in human brains, it has to be a structural constituent of the neuron, impacted by the neuropathological process, and easily detectable in blood. The composition of intermediate filament subunits in neurons varies depending on the nerve cell type and stage of development (Figure 1). At the earliest stage of embryonic development, neural stem cells express nestin (NES), a type VI intermediate filament protein that is down-regulated after differentiation and replaced by cell type-specific intermediate filament proteins (Lendahl et al., 1990). Vimentin (VIM), a type III intermediate filament protein of mesenchymal cells, is also transiently co-expressed with nestin in precursor nerve cells (Yabe et al., 2003). VIM is gradually replaced by peripherin (PRPH), alpha-internexin (INA), neurofilament medium chain (NfM), and NfL during embryonic development. Neurofilament heavy chain (NfH) chain expression is low in developing neurons and increases postnatally (Shaw and Weber, 1982; Pachter and Liem, 1984).

Mature mammalian neurons usually express five different NfPs: NfL, NfM and NfH chains, as well as INA and PRPH. In mature neurons in the CNS, Nfs are generally composed of NfL, NfM, NfH, and INA (Yuan et al., 2006), whereas, in the peripheral nervous system, they mainly consist of NfL, NfM, NfH and PRPH (Yuan et al., 2012). Like all IF proteins, NfPs all share a common alpha-helical rod domain that assembles to form a filament backbone, flanked by variable amino and carboxy-terminal domains that regulate polymer assembly and interactions. NF heteropolymer assembly starts with the formation of NfP dimers and antiparallel aggregation of these dimers leads to formation of tetramers which are thought to be the basic subunit of NFs during assembly (Mucke et al., 2018) and usually consist of NfL and one or more of the other Nf proteins. NfPs of mature neurons *in vivo* are mainly stable polymers and the pool of soluble NfP is small.

Neurofilament proteins are mainly synthesized in the cell body and transported as hetero-oligomeric assemblies and short filaments into axons and dendrites (Pachter and Liem, 1984; Yuan et al., 2003, 2009; Yan and Brown, 2005) to establish a highly stable regionally specialized NF network (Nixon and Logvinenko, 1986; Nixon et al., 1994; Sanchez et al., 1996). Nf mRNAs are also transported out of cell bodies into dendrites, spines, and axons and localized NfP synthesis in these cytoplasmic extensions is used to spatially and temporally regulate their protein content in these subcellular domains (Alami et al., 2014). NfPs can be proteolyzed by calpains, the proteasome, and autophagy into many smaller degradation products (Yuan et al., 2017).

### The Neuropathological Basis for Neurofilament Proteins as Biomarkers

Biochemical, genetic, and animal model evidence implicates NfPs as a pathogenic culprit playing primary or secondary roles in nervous system diseases. NfPs are involved in the pathophysiological processes underlying many states of neurological injury and neurodegeneration, reflecting changes in structural integrity and abnormal accumulation or maldistribution of NfPs (Hamberger et al., 2003).

#### Animal Studies

Proper levels of NfPs are important for the normal functions of nervous systems in animals. Absence of NfL from neurons reduces axon diameters and causes sensorimotor and cognitive impairments in quails (Yamasaki et al., 1991) and mice (Zhu et al., 1997; Yuan et al., 2018). Single deletion of NfM, NfH or PRPH in mice can lead to age-related atrophy of motor axons (Elder et al., 1999), decrease in conduction velocity (Kriz et al., 2000) and reduced numbers of unmyelinated sensory axons (Lariviere et al., 2002), respectively. Deletion of INA in the absence of NfL (Yuan et al., 2003) or both NfL and NfH results in reduced transport of NfM into axons (Yuan et al., 2015b). Overexpression of NfL, NfM, NfH or PRPH in animals can produce neuropathology of motor neuron diseases (Cote et al., 1993; Xu et al., 1993; Beaulieu et al., 1999; Gama Sosa et al., 2003) while overexpression of INA leads to motor coordination deficits (Ching et al., 1999). In addition to the importance of NfP levels, expression of an NfL mutation in mice which causes human disease (Zuchner et al., 2004; Filali et al., 2011; Liu et al., 2011; Shen et al., 2011; Pisciotta et al., 2015) also leads to motor neuropathology (Lee et al., 1994) and phenotype of CMT (Filali et al., 2011) probably due to disruption of Nf assembly (Perez-Olle et al., 2002; Tradewell et al., 2009) and transport (Brownlees et al., 2002), and abnormal Nf accumulation (Zhai et al., 2007).

#### Human Studies

**FIGURE 2:**
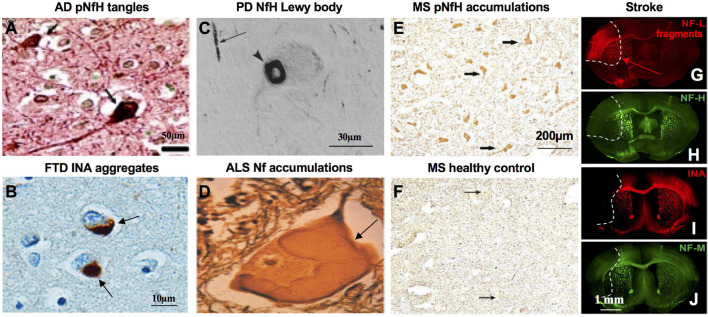
Pathological basis of NfPs as biomarkers in neurologic diseases and neuronal injury. **(A)** NFTs in AD brain are stained with pNfH with mouse monoclonal phospho-NfH antibody RT97 under the condition it does not cross-react with phosphor-tau (adapted from Rudrabhatla et al., 2010). **(B)** Cytoplasmic inclusions in NIFID brain, a type of FTD, are stained with antibody to alpha-internexin (adapted from Cairns et al., 2004). **(C)** Cytoplasmic Lewy bodies in PD brain are stained with antibody to NfH (adapted from Goldman et al., 1983). **(D)** Masses of Nf swelling in ALS spinal cord are stained with Silver (adapted from Cleveland and Rothstein, 2001). **(E)** Anterior horn cell perikarya in MS spinal cord are prominently stained with antibody to pNfH (SMI31) whereas healthy controls remain almost non-reactive **(F)** (adapted from Muller-Wielsch et al., 2017). Ischemia-affected areas in mouse brain 24 h after experimental stroke induction are demarcated by an increase of NfL degradation fragments immunoreactivity **(G)**, while the immunosignals for NfH **(H)**, alpha-internexin (INA) **(I)**, and NfM **(J)** are decreased (adapted from Mages et al., 2018).

Clinical studies demonstrate presence, normal structure and assembled network of NfPs are critical for human health. NfL loss of function mutations in cases of human neuropathy which cause markedly lowered NfL protein levels reduce axon diameters and cause sensorimotor and cognitive impairments in humans (Yum et al., 2009; Sainio et al., 2018). NfL and NfH mutations can cause Nf accumulation in CMT type 2E/1F/CMTDIG (Lerat et al., 2019) and CMT2CC (Ikenberg et al., 2019). In AD, NfPs are integral components of neurofibrillary tangles (Rudrabhatla et al., 2011; Figure 2) and NfH and NfM are 4–8-fold more phosphorylated than normal (Rudrabhatla et al., 2010). In PD, Lewy bodies contain NfPs (Goldman et al., 1983) and a cage-like Nf structure encapsulates Lewy bodies (Moors et al., 2019). In Nf inclusion disease, a form of FTD, prominent aggregations of NfPs, especially INA, are the neuropathologic hallmark of the condition (Cairns et al., 2004). Abnormal NfP accumulations are also a hallmark pathologic feature of ALS (Cleveland and Rothstein, 2001). In MS, increased expression of phosphorylated NfH (pNfH) is observed in spinal motor neuron perikarya (Muller-Wielsch et al., 2017) and Nfs accumulate excessively in axons in GAN (Bomont et al., 2000).

### Neurofilament Proteins Released From Neurons Gain Access to Blood Under Physiological and Pathological Conditions

#### Recent Technology Breakthroughs for the Reliable Detection of Neurofilament Proteins in the Peripheral Circulation

Low levels of NfPs are constantly released from neurons into CSF and blood under physiological conditions and rise above normal in pathological states.

Rosengren et al. (1996) first tested NfPs as possible biomarkers using enzyme-linked immunosorbent assay (ELISA) with polyclonal rabbit antisera specific against the individual NfPs and showed that CSF NfL levels were increased in patients with ALS and AD compared to controls. However, the sensitivity of ELISA and the later developed electrochemiluminescence (ECL) immunoassay does not allow small, disease-related changes to be reliably detected in peripheral circulation. In 2010, single-molecule enzyme-linked immunosorbent assay (Simoa) was initially described (Rissin et al., 2010) which later enabled reliable quantification of NfL in serum or plasma samples (Gisslen et al., 2016) using NfL-specific monoclonal antibodies (mAb47:3) (Norgren et al., 2002). More recently, Meso Scale Discovery, immunomagnetic reduction technologies and the Ella platform based on microfluidic channels have also been developed to detect low NfP levels in blood (Liu H.C. et al., 2020; Lombardi et al., 2020; Gauthier et al., 2021).

#### Neurofilament Proteins in Exosomes

**FIGURE 3:**
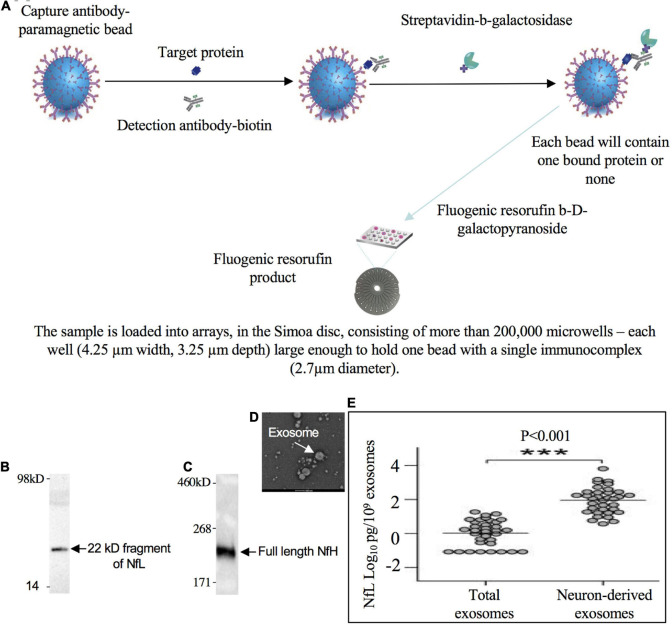
22 kD fragment of NfL and full length NfH in blood. **(A)** Low levels of NfPs in blood can be detected with single molecule array technology (Simoa/digital ELISA). A 22 kDa degradation fragment of NfL (**B**, adapted from Lombardi et al., 2020) and full length NfH (adapted from Adiutori et al., 2018) were detected in blood **(C)**. **(D)** Isolated exosomes from blood (adapted from Zhang et al., 2020). **(E)** NfL signal is enriched in neuron-derived exosomes compared to total or astrocyte-derived exosomes in blood (adapted from Sun et al., 2017). *** Indicates highly significant.

The fact that plasma NfL levels are enriched in neuron-derived exosomes compared to total exosomes isolated from blood in healthy individuals (Sun et al., 2017) suggests the NfPs are released from neurons at least in the form of exosomes (Figure 3). Moreover, plasma neuron-derived exosomes contain about 74-fold more NfL than plasma astrocyte-derived exosomes, which have only negligible amounts (Winston et al., 2019). NfP-containing exosomes or NfPs or degradation fragments released into the extracellular space may be eliminated from the CNS along intramural peri-arterial drainage pathway (Engelhardt et al., 2017).

#### Neurofilament Protein Forms in Peripheral Circulation

Identity of the NfL forms in plasma exosomes is still unclear but a 22 kDa NfL degradation fragment has been revealed with an anti-NfL antibody and shown to be increased in ALS patients (Lombardi et al., 2020). Also identified are a 30 kDa fragment of NfL in Nf-containing aggregates from human blood (Adiutori et al., 2018) and a 10 kDa fragment of NfL in mouse CSF. Since no full length NfL has been ever reported in CSF or blood (Brureau et al., 2017), the detected Simoa signal is, therefore, NfL immunoreactivity or NfL breakdown product. By contrast, full length (200 kDa) or oligomeric NfH were predominant in CSF and blood (Petzold et al., 2003; Shaw et al., 2005; Lewis et al., 2008). Recent studies also suggest full length (150 kDa) or trimeric NfM (450 kDa) in blood (Haggmark et al., 2014). A comprehensive list of widely used capture and detection antibodies to NfPs in ELISA is shown in Table 1.

**TABLE 1:** NfP and fragment measurement as biomarkers.

	Capture antibodies	Detection antibodies	References
**ELISA for measuring Nf subunits as biomarkers**			
NfL	Chicken polyclonal anti-NfL	Rabbit polyclonal anti-NfL	Rosengren et al., 1996
	NfL mAb 47:3, core domain (aa92-396)	NfL mAb 2:1	Norgren et al., 2003
	Polyclonal antibody R61d to NfL, NfM and NfH	NfL mAb NR4	Hu et al., 2002
	NfL 21 mAb, core domain (aa93-396)	NfL 23 mAb	Gaetani et al., 2018
NfH	SMI31 mAb anti-NfH and NfM	Anti-NfH NA211	Hoglund et al., 2012
	SMI34 mAb anti-NfH	Rabbit polyclonal anti-NfH	Zucchi et al., 2018
	SMI35 mAb anti-NfH	Chicken polyclonal anti-NfH	Petzold et al., 2003
	Chicken polyclonal anti-NfH	Rabbit polyclonal anti-NfH	Shaw et al., 2005
	AH1 mAb anti-NfH	NAP4 mAb anti-NfH	Boylan et al., 2009
	Polyclonal antibody R61d to NfL, NfM and NfH	SMI31, SMI32, SMI33 and SMI34	Hu et al., 2002
	pNfH mAb 9C9	NfH polyclonal antibody	Koel-Simmelink et al., 2014
NfM	Polyclonal antibody R61d to NfL, NfM and NfH	SMI31, SMI32 and SMI33	Hu et al., 2002
	Monoclonal anti-NfM	Monoclonal anti-NfM	Martinez-Morillo et al., 2015
	Polyclonal anti-NfM	Polyclonal anti-NfM	Zucchi et al., 2018
PRPH	Rabbit polyclonal anti-PRPH	Chicken polyclonal anti-PRPH	Finderlater, 2010
	Unknown	Unknown	Sabbatini et al., 2021
**Proteomics for measuring NF subunits as biomarkers**			
NfM			Haggmark et al., 2014; Martinez-Morillo et al., 2014; Remnestal et al., 2020
INA			Martinez-Morillo et al., 2014
PRPH			Liang et al., 2019

#### Neurofilament Light Chain Levels in Normal Individuals

Intracellular NfPs have long half-lives ranging from 55 days in axons (Nixon and Logvinenko, 1986; Yuan et al., 2015a) to 64–72 days at synapses (Heo et al., 2018), indicating their slow turnover rates inside neuronal compartments. Upon release into the extracellular space, serum or plasma NfL levels in healthy individuals are about 2.5% of the levels in CSF and correlate highly with the 40-fold higher NfL concentrations in CSF with typical R values ranging from 0.6 to 0.7 (Disanto et al., 2017; Pereira et al., 2017; Khalil et al., 2020; Alagaratnam et al., 2021), suggesting that most of the NfL signal in blood is CNS-derived and could be used as a proxy measure for CSF NfL levels (Gisslen et al., 2016). The NfL levels in blood are most often tested in serum and less frequently in EDTA-plasma with serum levels slightly higher than in plasma (Hviid et al., 2020b). Either specimen type is acceptable, however, when used in either research or clinical setting a single specimen should be selected for use. Plasma NfL levels measured in the morning may be more than 10% higher than those measured in the evening, suggesting that synaptic remodeling during sleep might alter NfL kinetics (Benedict et al., 2020; Thebault et al., 2021). CSF NfL levels in healthy females are about 20% lower than levels in healthy males (Bridel et al., 2019) although the reverse was true in an ALS cohort (Thouvenot et al., 2020). Concentrations of CSF and serum NfL increase with age in healthy controls (Yilmaz et al., 2017) with an increase in adult control serum NfL levels of 2.2% per year of age (Disanto et al., 2017; Barro et al., 2018). These increases accompany hippocampal atrophy in cognitively healthy older adults, which has suggested possible AD-independent, age-expected hippocampal decline (Idland et al., 2017). However, younger children have higher serum NfL levels than older children reaching the lowest level between the age of 10 and 15 years, then increasing in a linear fashion until the age of 60 years and accelerating non-linearly afterward (Evers et al., 2020; Khalil et al., 2020; Reinert et al., 2020). There are various proposed bases for serum NfL elevation in aging, including subclinical senescence with greater neuronal apoptosis (Khalil et al., 2020) and increased disruption of blood-brain barrier (Sweeney et al., 2018). Levels of serum NfL may also be affected by race, systolic blood pressure, decreased renal function, glycemic control measured by hemoglobin A1C (Korley et al., 2019) and pregnancy (Cuello et al., 2019). The multiplicity of influences on these levels prompts caution in controlling stringently for confounding variables in clinical studies.

#### Contribution of Neurofilament Proteins or Fragments From Different Neuronal Compartments

Besides calpains, the proteasome and autophagy (Smerjac et al., 2018), other non-specific proteases, including cathepsin D (Nixon and Marotta, 1984) and caspases 6 and 8 (Shabanzadeh et al., 2015) can also trigger Nf turnover and generate Nf peptides. Nf assembly confers significant proteolytic resistance to Nf subunits: deletion of three Nf subunits leads to degradation of the fourth subunit (Yuan et al., 2015b). Phosphorylation also protects Nfs against proteolysis (Goldstein et al., 1987; Pant, 1988; Rao et al., 2012). NfPs or their degradation fragments are released into biofluids following any damage to nervous system. Therefore, they are neither able to determine brain region specific alterations nor differentiate disease specific pathophysiological process.

#### Mechanisms for Neurofilament Protein and Fragment Release From Neurons

The exact mechanisms governing NfP release into biofluid are not fully understood. Release of NfPs or fragments from neurons may be a direct passive consequence of the loss of membrane integrity or may follow the known pathways for active secretion of other neuronal peptides and proteins. Intracellular endosomal organelles known as multivesicular bodies may play critical roles in the release of peptides (Von Bartheld and Altick, 2011). This may happen though “back-fusion” events and budding from the plasma membrane to generate micro-vesicles (Kleijmeer et al., 2001) or through release of smaller endosomally derived exosomes (Lachenal et al., 2011). Levels of NfL signals in isolated neuron-derived exosomes accounting for a small percentage of total NfL concentration in plasma suggest that active secretion is at least one of the mechanisms for NfP release from neurons. After release from neurons, some NfPs and fragments can be degraded and cleared by varied extracellular proteinases and microglia and these processes may even further generate the fragments from a larger form. Pathways for degradation could be differentially critical in the context of healthy, injured or chronically damaged neurons. Expression of NfP genes is not elevated in ALS (Wong et al., 2000) and neither NfP gene (Robinson et al., 1994) nor protein expression (Ashton et al., 2019) is elevated in AD, suggesting that the increased NfP signal in biofluids is not due to a compensatory overproduction.

#### Major Determinants of Neurofilament Protein and Fragment Levels in Cerebrospinal Fluid and Blood

Studies have linked NfP levels in blood to changes in white matter (Moore et al., 2018; Spotorno et al., 2020; Maggi et al., 2021), gray matter (Jakimovski et al., 2019a; Kang et al., 2020), or both (Johnson et al., 2018), yielding a confusing picture of what variables dictate the highly variable levels found in different disorders. Some likely determinants of blood/CSF levels, however, include the composition of the diseased or injured area (relative abundance of large caliber axons that have high Nf-content) and size of the damaged region. NfL and NfH content in spinal cord is several fold higher than in corpus callosum (Yuan et al., 2012) and at least 10-fold higher than in cortex (Shaw et al., 2005). Accordingly, a spinal cord injury released about 12-fold more NfH into blood than a brain injury of comparable size (Shaw et al., 2005). Demyelinating damage to CNS axons associated with clinical or MRI (magnetic resonance imaging) disease activity in MS can cause a spike of more than 20-fold in the levels of serum NfL which may be lowered with effective treatment (Akgun et al., 2019). Only about 20% of the NfL fragment present in blood comes from neuron-derived exosomes (Altick et al., 2009; Winston et al., 2019; Guedes et al., 2020) so the extent of loss of membrane integrity affecting NF-rich axons, or even to a lesser extent synapses, is likely the major determinant of NfP and fragment levels in CSF and blood. In a limited region of involvement as in the substantia nigra pars compacta in PD, NfL fragment level increases in CSF and serum are modest. By contrast, in FTD/ALS, widespread degeneration of large caliber Nf-rich axonal fibers in the spinal cord and brain results in one of the highest elevations of NfP and fragment blood levels among neurodegenerative diseases. More studies are warranted to determine the relative gray and white matter contributions to NfP and fragment levels in biofluids at different stage of a specific disease.

#### Mechanisms of Neurofilament Protein or Peptide Trafficking Between Brain and Blood

Since the main source of serum NfPs is the CNS, it is not fully clear how NfPs traffic between parenchymal, CSF and blood compartments. NfPs or their degradation fragments could also follow the apparent general pathways by which molecules such as amyloid-beta peptides pass from the interstitial fluid (ISF) of the brain into CSF and blood. Soluble metabolites or peptides from cells in most organ are absorbed directly into the blood or drain via lymphatic vessels to regional lymph nodes (Engelhardt et al., 2017). Soluble tracers such as serum albumin injected into ISF of the brain drain to cervical lymph nodes along the walls of cerebral arteries (Szentistvanyi et al., 1984) through intramural peri-arterial drainage pathway (IPAD) (Albargothy et al., 2018) including initially along basement membranes that surround capillaries and then along the basement membranes between smooth muscle cells in the tunica media of intracerebral and leptomeningeal arteries (Carare et al., 2008). About 85% of a tracer injected into the cerebral hemispheres passes to cervical lymph nodes via IPAD (Szentistvanyi et al., 1984) while only 10–15% passes into the CSF (Szentistvanyi et al., 1984; McIntee et al., 2016). Future studies need to measure the proportion of NfPs and degradation fragments released from neurons that reaches the CSF. Drainage of CSF into lymphatic vessels of the nasal mucosa via the cribriform plate appears to be a major lymphatic drainage pathway (Kida et al., 1993; De Leon et al., 2017) and may also include dural lymphatics (Aspelund et al., 2015). The glial-lymphatic or glymphatic pathway is recently identified in rodent brain, which sub-serves the flow of CSF into the brain along perivascular spaces and then into the brain interstitium facilitated by aquoporin-4 water channels (Rasmussen et al., 2018). This pathway then directs flow toward the venous perivascular and perineuronal spaces, ultimately clearing solutes from neuropil into meningeal and cervical lymphatic drainage vessels.

#### Dynamics of Extracellular Neurofilament Proteins and Fragments

In acute neurological diseases with a known timepoint for neuronal or axonal damage such as traumatic brain injury (TBI) (Bergman et al., 2016; Shahim et al., 2017), stroke (Gattringer et al., 2017; Tiedt et al., 2018) and MS (Rosso et al., 2020), CSF and serum NfP signals increased over a few days and remained elevated over many months. It may be difficult to investigate the dynamics of CSF and serum NfPs in chronic neurodegenerative diseases such as AD and PD without treatments that can cure them. Because it is suggested that serum NfL fragments may be cleared by the kidneys, renal function ought to be considered when interpreting serum NfL levels (Korley et al., 2019; Van Der Plas et al., 2021).

### Neurofilament Proteins as Biomarkers in Animal Models

#### Neurofilament Proteins as Biomarkers in Animal Models of Neurological Diseases

Increased levels of plasma NfL have been observed in mouse models of PD A53T- alpha-synuclein, tauopathy P301S-Tau and AD APP/PS1 (amyloid precursor protein/presenilin 1) (Bacioglu et al., 2016). Increases in NfL in CSF and blood coincide with the onset and progression of the corresponding proteopathic lesions in brain. Experimental induction of alpha-synuclein lesions increase blood NfL levels, while blocking the development of amyloid-beta lesions attenuates NfL increases (Bacioglu et al., 2016). Prolonged expression in mice of p25 (the calpain-mediated truncated product of p35, the regulatory subunit of Cdk5 – cyclin-dependent-like kinase 5) causes severe synaptic and neuronal loss and brain atrophy which are accompanied by cognitive deficits (Fischer et al., 2005). In these inducible CamKII-TetOp25 transgenic mouse models of neurodegeneration, serum NfL levels increase after induction of neurodegeneration by switching on p25 transgene expression via removal of doxycycline but do not increase further if induction is stopped by switching off p25 expression. Increased levels of serum NfL correlate with induced neuronal damage in the cortex and hippocampus of CamKII-TetOp25 mice, indicating that NfL levels mirror the ongoing neurodegeneration and neuronal loss and may be used as a dynamic biomarker of neurodegeneration (Brureau et al., 2017). In HD R6/2 mice, increased levels of NfL in CSF and serum is associated with neurodegeneration and disease severity (Soylu-Kucharz et al., 2017). In 304Q knock-in spinocerebellar ataxia type 3 (SCA3) mouse model, serum NfL and pNfH are elevated at the pre-symptomatic stage of 6 months of age and correlate with ataxin 3 aggregation and Purkinje cell loss in the brain (Wilke et al., 2020). Increased CSF pNFH levels were also observed in horses with equine neuroaxonal dystrophy/degenerative myeloencephalopathy (Edwards et al., 2021). Plasma pNfH levels also closely reflect later stages of disease progression and therapeutic response in the SOD1 (superoxide dismutase 1) G93A mouse model of ALS (Lu et al., 2012). Recently, serum NfL concentration in sheep with prion disease was more than 15 times higher than that found in control samples (Zetterberg et al., 2019). More recently, plasma NfL levels were also reported to reflect disease severity in mice inoculated with prions and fell significantly in antisense oligonucleotide-treated mice compared to the immediate pre-dose timepoint, suggesting a reversal of pathology driving the 53% increase in survival time (Minikel et al., 2020).

#### Neurofilament Proteins as Biomarkers in Animal Models of Neurological Injuries

Following experimental spinal cord injury (SCI) in adult rats, serum pNfH showed an initial peak of expression at 16 h and a second peak at 3 days while no serum pNfH is detectable in sham control animals (Shaw et al., 2005). The maximum level of pNfH in these SCI experiments was 250 ng/ml pNfH in the 3–5 day post-injury period following injury. Serum pNfH showed a similar trajectory in TBI in adult rats but the average peak level of expression of serum pNfH was only about 20 ng/ml, much lower than that seen in the SCI model (Shaw et al., 2005). Recent studies found serum NfL levels were substantially elevated at all acute and subacute time-points after a single mild TBI (mTBI), peaked at 1-day, and remained elevated 14-days post-injury (O”Brien et al., 2021). Increased serum NfL levels were also mTBI dose-dependent and correlated with the degree of sensorimotor impairment (O”Brien et al., 2021). In more recent studies using an experimental rat model of blast-induced TBI, pNfH levels increased at 24 hr, returned to normal levels at 1 month, but increased again at 6 months and 1 year post-blast exposure (Arun et al., 2021). Moreover, the changes in CSF pNfH correlate with pNfH levels in brain regions and with neurobehavioral function in the rats (Arun et al., 2021).

### Neurofilament Proteins as Biomarkers in Neurological Diseases

Cerebrospinal fluid or serum NfL and pNfH have been widely studied as biomarkers in a number of neurological diseases (Table 2) or conditions directly or indirectly affecting central and peripheral nervous systems (Table 3). NfPs are not only elevated in neurological diseases but may also track disease progression. Different subunits might reflect different neurodegenerative processes. In addition to commonly used NfL and pNfH, some studies also found potential values of other Nf subunits, i.e., NfM (Hu et al., 2002; Haggmark et al., 2014; Martinez-Morillo et al., 2014; Zucchi et al., 2018; Remnestal et al., 2020), INA (Martinez-Morillo et al., 2014) and PRPH (Finderlater, 2010; Liang et al., 2019; Sabbatini et al., 2021) as biomarkers in CSF or serum in neurological diseases or injuries.

**TABLE 2:** NfPs and fragments as biomarkers in neurodegeneration and neuronal injuries.

Neurological diseases and injuries	Association of NF subunit level with								Association of NF subunit level with			References
	NfL		pNfH (smi35)		NfH (smi34)		NfH (smi31 and others)					
	B	C	B	C	B	C	B	C	Disease activity	Prognosis	Treatment response	
Multiple sclerosis and clinically isolated syndrome	+	+	+	+	+	+	+	+	yes	yes	yes	Linker et al., 2009; Disanto et al., 2016, 2017; Herrera et al., 2019; Calabresi et al., 2020; Saraste et al., 2021
Alzheimer’s disease	+	+		+			+	+	yes	yes	yes	Rosengren et al., 1996; Hu et al., 2002; Kuhle et al., 2010; Hoglund et al., 2012; Zetterberg et al., 2016; Mattsson et al., 2017; Gaetani et al., 2018; Benedet et al., 2020
Adult Down syndrome	+								yes	yes		Fortea et al., 2018; Strydom et al., 2018; Shinomoto et al., 2019; Delaby et al., 2020; Carmona-Iragui et al., 2021; Petersen et al., 2021
Mild cognitive impairment	+	+							yes	yes		Zhou et al., 2017; Mayeli et al., 2019; Osborn et al., 2019
Vascular dementia		+				+		+	yes	yes		Hu et al., 2002; Skillback et al., 2014
Mixed dementia		+							yes	yes		Skillback et al., 2014
Frontal temporal dementia		+							yes	yes		Skillback et al., 2014; Remnestal et al., 2020
Dementia with Lewy body				+								De Jong et al., 2007
HIV-associated dementia	+								yes	yes	yes	Gisslen et al., 2016
Stroke	+	+		+					yes	yes		Norgren et al., 2003; Petzold et al., 2003; Kuhle et al., 2010; Martinez-Morillo et al., 2014; Tiedt et al., 2018; Garland et al., 2021; Peng et al., 2021; Wang Z. et al., 2021
Traumatic brain injury	+								yes	yes		Shahim et al., 2016; Liang et al., 2019; Yang et al., 2019
Sport-related concussion	+											Shahim et al., 2017; McDonald et al., 2021
Spinal cord injury	+						+		yes	yes		Shaw et al., 2005; Kuhle et al., 2015
Amyotrophic lateral sclerosis	+	+	+	+			+	+	yes	yes		Rosengren et al., 1996; Kuhle et al., 2010; Haggmark et al., 2014; Rossi et al., 2018; Benatar et al., 2019
Parkinson’s disease	+	+							yes	yes		Lin et al., 2018, 2019; Backstrom et al., 2020; Ye et al., 2021
Huntington disease	+								yes	yes		Byrne et al., 2017; Rodrigues et al., 2020
Bipolar disorder		+										Jakobsson et al., 2014
Autism spectrum disorder	+											He et al., 2020
Neuronal ceroid lipofuscinosis type 2 and 3	+										yes	Ru et al., 2019; Dang Do et al., 2020
Spinal muscular atrophy	+								yes	yes	yes	Olsson et al., 2019
Cortico-basal degeneration	+											Hansson et al., 2017
Multiple system atrophy	+											Hansson et al., 2017
Progressive supranuclear palsy	+											Hansson et al., 2017
Spinocerebellar ataxia	+								yes	yes		Li et al., 2019; Coarelli et al., 2021
Friedreich ataxia	+								yes			Clay et al., 2020
Epilepsy								+				Rejdak et al., 2012
Charcot-Marie-Tooth disease	+								yes			Sandelius et al., 2018; Millere et al., 2021
Hereditary transthyretin amyloidosis	+								yes	yes	yes	Kapoor et al., 2019; Ticau et al., 2019
Guillain-Barre syndrome	+			+								Kuhle et al., 2010; Mariotto et al., 2018
Chronic inflammatory demyelinating polyneuropathy	+								yes			Hayashi et al., 2021
Neuromyelitis optica				+								Miyazawa et al., 2007; Liu et al., 2021
Creutzfeldt-Jacob disease (prion disease)	+								yes	yes	yes	Steinacker et al., 2016; Minikel et al., 2020; Thompson et al., 2021
Canine cognitive dysfunction syndrome	+								yes			Vikartovska et al., 2020

*B, blood; C, CSF.*

**TABLE 3:** NfPs and fragments as biomarkers in conditions affecting nervous system.

Conditions	Association of NF subunit level with						Association of NF subunit level with			References
	NfL	pNfH (smi35)			NfH (smi31 and others)					
	B	C	B	C	B	C	Disease activity	Prognosis	Treatment response	
Acute bacterial meningitis	+									Gronhoj et al., 2021
Anesthesia and surgery	+									Evered et al., 2018
Anorexia nervosa	+									Nilsson et al., 2019
Autoimmune encephalitis		+		+					yes	Kortvelyessy et al., 2018; Fominykh et al., 2019; Piepgras et al., 2021
Brain metastasis and glioma	+						yes	yes		Hepner et al., 2019
Cardiac arrest	+						yes	yes		Moseby-Knappe et al., 2019
Cerebral small vessel disease	+						yes	yes		Egle et al., 2021; Qu et al., 2021
Chemotherapy-induced cognitive impairment					+		yes			Natori et al., 2015
Chorea-acanthocytosis	+						yes			Peikert et al., 2020
Diabetic neuropathy					+					Qiao et al., 2015
Hypoxic-ischemic encephalopathy						+	yes			Douglas-Escobar et al., 2010
Idiopathic normal pressure hydrocephalus		+								Jeppsson et al., 2013
Intrapartum asphyxia	+						yes			Toorell et al., 2018
Mcleod syndrome	+									Peikert et al., 2020
Mitochondrial encephalomyopathy, lactic acidosis, and stroke-like episodes	+						yes			Zheng et al., 2021
Mitochondrial encephalopathy		+					yes	yes		Sofou et al., 2019
MOG-Abs-associated disorders				+						Sara et al., 2021
Neurosarcoidosis	+	+								Byg et al., 2021
Peri/intraventricular hemorrhage	+	+					yes	yes		Goeral et al., 2021
Postoperative delirium	+									Casey et al., 2019
Preeclampsia	+						yes	yes		Evers et al., 2018
Preterm infants	+									Depoorter et al., 2018
Sepsis-associated encephalopathy	+						yes	yes		Ehler et al., 2019
Severe COVID-19	+									Sutter et al., 2021
Thoracolumbar intervertebral disk herniation					+		yes	yes		Nishida et al., 2014
Wilson’s disease	+						yes			Shribman et al., 2021
X-linked adrenoleukodystrophy	+						yes		yes	Weinhofer et al., 2021

*B, blood; C, CSF.*

#### Multiple Sclerosis

Patients with MS have up to 60% axonal loss at all spinal levels involving all fibers regardless of their diameter (Tallantyre et al., 2009; Petrova et al., 2018). The concentrations of CSF and serum NfPs represent the degree of axonal loss and therefore, could be a biomarker of MS disease activity. Accordingly, CSF NfL levels in relapsing MS were 3-fold higher than in healthy controls (951.8 vs. 284.4 pg/ml) and associated with relapse and cortical lesions (Damasceno et al., 2019). Serum NfL levels was first reported to be increased in early relapsing MS and correlated with MRI measures of disease severity using an electro-chemiluminescence assay (Kuhle et al., 2016). This finding of serum NfL as a biomarker of MS disease activity was later substantiated with higher sensitivity Simoa digital immunoassay (Hansson et al., 2017; Novakova et al., 2017; Kuhle et al., 2019; Szilasiova et al., 2021). Increased levels of serum NfL are also associated with MS brain T2 lesion load (Disanto et al., 2017). A recent study showed that serum NfL levels were associated with T1, T2 and gadolinium-enhancing lesion volumes at baseline and higher serum levels of NfL at baseline were associated with greater atrophy of the whole brain, gray matter and deep gray matter nuclei in the long term (Jakimovski et al., 2019a). Serum NfL may also detect MS disease activity that escapes detection in routine MRI (Akgun et al., 2019). The levels of serum NfL increased 6 years before clinical MS onset, indicating MS may have a prodromal phase lasting several years and that neuronal damage occurs already during this phase (Bjornevik et al., 2019). After clinical onset, a 1-point Expanded Disability Status Scale (EDSS) increase corresponds to an serum NfL increase of about 14% (Disanto et al., 2017).

Serum NfL concentrations have been used to assess disease progression in MS. Clinically isolated syndrome (CIS) is one of the MS disease courses and refers to a first episode of neurological symptoms that last at least 24 h and is caused by inflammation or demyelination of in the CNS. Elevated NfL levels in both pediatric and adult patients with CIS have been reported to be associated with a shorter time to clinically definite MS diagnosis independent of other prognostic factors (Van Der Vuurst De Vries et al., 2019). In patients with confirmed relapsing or progressive MS, baseline serum NfL can predict short-term outcomes including clinical and cognitive performance (Disanto et al., 2017; Jakimovski et al., 2019b; Filippi et al., 2020). Serum NfL levels sampled within the first 5 years of MS symptom onset was shown to independently predict long-term worsening EDSS score and risk of developing progressive MS in patients followed longitudinally for 15–26 years (Thebault et al., 2020a). Notably, patients with serum NfL levels less than 7.62 pg/ml were 7.1 times less likely to develop progressive MS (Thebault et al., 2020a).

Serum/plasma or CSF NfPs have potential utility for assessing treatment efficacy in single patients and beginning at an earlier stage in the disease course. Treatment with any disease-modifying therapy in MS has been reported to be associated with significantly lower serum NfL levels compared to untreated individuals (Disanto et al., 2017; Harris et al., 2021), proving that CSF or serum/plasma NfL is a therapeutic response biomarker in MS that may be related to consequent prevention of ongoing neuronal damage.

Fingolimod significantly reduced plasma NfL levels after 6 months and until the end of the studies (24 months) (Kuhle et al., 2019). Similarly, CSF NfL and NfH^*SMI*35^ levels were significantly lowered after 12 months of natalizumab treatment. A 4 fold greater reduction of NfL than of NfH^*SMI*35^ suggests differential sensitivity to therapeutic changes using different subunits as the biomarker (Kuhle et al., 2013) although NfH^*SMI*35^ antibodies detect NfH phosphorylation rather than the protein itself and may reflect different aspects of a given disease. Caution should be taken when MS patient are at risk for other treatment-induced neurological complications that can cause serum NfL levels to rise, such as natalizumab-induced progressive multifocal leukoencephalopathy (Dalla Costa et al., 2019) and ablative hemopoietic stem cell transplantation (Thebault et al., 2020b).

#### Amyotrophic Lateral Sclerosis

Mutation carriers with ALS symptoms have higher NfPs than those without ALS symptoms (CSF NfL 37-fold, 7388 vs. 195.7 pg/ml) (Weydt et al., 2016), suggesting that elevated NfP levels are linked to disease progression and the symptomatic disease phase (Benatar et al., 2019; Gille et al., 2019). Moreover, elevated serum NfL levels were observed as far back as 1 to 3.5 years before symptom onset depending on different gene mutations (SOD1, 12 months; FUS, 2 years and C9orf72, 3.5 years) (Benatar et al., 2018, 2019). CSF NfL levels also correlate with the extent of upper motor neuron and lower motor neuron involvement in ALS (Poesen et al., 2017). The time to generalization in ALS is an early clinical parameter of disease progression and CSF NfL concentrations have been shown to predict the conversion from bulbar/spinal to generalized ALS (Tortelli et al., 2015). Levels of NfL and pNfH also correlate with survival length in ALS (Brettschneider et al., 2006; Zetterberg et al., 2007; Lu et al., 2015). Higher serum NfL at diagnosis is also one of several factors that predict time of death in ALS (Thouvenot et al., 2020). In a recent clinical trial, levels of pNfH and NfL in plasma and CSF were largely unchanged in placebo-treated patients due to superoxide dismutase 1 (SOD1) mutations and decreased in patients treated with tofersen administered intrathecally over a period of 12 weeks, an antisense oligonucleotide that mediates the degradation of SOD1 messenger RNA to reduce SOD1 protein synthesis (Miller et al., 2020). Moreover, CSF SOD1 concentration decreased in these tofersen-treated patients with evidence of a slowing in the disease in the total scores on the ALS functional rating scale and the handheld dynamometry megascore.

#### Alzheimer’s Disease

Plasma NfL is significantly higher in patients with MCI (mild cognitive impairment) (42.8 pg/ml) and patients with AD (51.0 pg/ml) compared with healthy controls (34.7 pg/ml) (Mattsson et al., 2017). This finding was further confirmed by other studies (Zhou et al., 2017; Lewczuk et al., 2018). Moreover, higher NfL levels were associated with cognitive decline in non-dementia older adults (He et al., 2021). Interestingly, elevated plasma NfL is associated with the presence of amyloid-beta plaques in pre-symptomatic individuals whereas NfL levels is associated with the load of tau in symptomatic patients (Benedet et al., 2020). Plasma NfL is also associated with AD progression independent of amyloid-beta (Moscoso et al., 2021a). Plasma NfL levels also correlate with Braak staging and longitudinal increases in plasma NfL are observed in all Braak groupings (Ashton et al., 2019). In addition, normal plasma NfL level (20.24 pg/ml) is also linked with resistance to PS1 familial AD in apolipoprotein E3 (APOE3) Christchurch mutation (Arboleda-Velasquez et al., 2019). The role of NfL as a potential biomarker for AD has been extensively reviewed and recent meta-analysis regarding its association with AD can be found elsewhere (Olsson et al., 2016; Khalil et al., 2018; Bridel et al., 2019; Jin et al., 2019). Recent studies further demonstrated plasma NfL levels or together with cognitive testing as predictors of fast progression (Santangelo et al., 2021) and future declines in cognition and function in AD (Li et al., 2021).

Hyperphosphorylation of tau neurofibrillary tangles is one of the hallmarks in AD. CSF ptau181 levels were first found to be increased significantly in patients with AD compared to healthy controls over two decades ago (Vanmechelen et al., 2000). This finding was subsequently verified by others (Lewczuk et al., 2004; Fagan et al., 2011; Tang et al., 2014) and later also was confirmed with the measurement of serum ptau181 (Shekhar et al., 2016). Recent studies demonstrated that blood ptau181 can predict cortical brain atrophy (Llibre-Guerra et al., 2019; Tissot et al., 2021), tau and amyloid-beta pathology (Lantero Rodriguez et al., 2020; Clark et al., 2021; Moscoso et al., 2021b), differentiate AD from other neurodegenerative diseases (Mielke et al., 2018; Thijssen et al., 2020; Grothe et al., 2021) and identify AD across the clinical continuum (Janelidze et al., 2020a; Karikari et al., 2020b, 2021). In familial AD, plasma ptau181 levels may rise as early as 16 years before clinical onset (O’connor et al., 2020). In addition to ptau181, some studies demonstrated ptau217 (Janelidze et al., 2020b; Karikari et al., 2020a; Palmqvist et al., 2020) and ptau231 are also useful biomarkers for AD (Kohnken et al., 2000; Suarez-Calvet et al., 2020; Ashton et al., 2021b). The combined use of these AD-specific biomarkers ptau181, ptau217, ptau231 with NfL as a disease-non-specific biomarker of neuronal integrity could improve prediction and monitoring of disease progression in AD (Moscoso et al., 2021a).

#### Frontotemporal Dementia

Serum NfL levels in patients with FTD were about 4-fold higher than in healthy controls (77.9 vs. 19.6 pg/ml) and the elevations correlate with disease severity (Rohrer et al., 2016). Moreover, increased serum NfL levels were observed 1 to 2 years before the clinical onset of symptoms (Van Der Ende et al., 2019), indicating pathophysiology of the disease in the preclinical phase.

#### Dementia With Lewy Bodies

Plasma NfL levels in patients with DLB were about 2-fold higher than in healthy controls (55.3 vs. 25.7 pg/ml) and the elevations correlate with disease severity and plasma NfL is the best predictor of cognitive decline compared to age, sex and baseline severity variables over a follow-up of 2 years (Pilotto et al., 2021).

#### Peripheral Neuropathy

Neurofilament proteins are most abundant in peripheral large-caliber myelinated axons such as sciatic nerves (Hoffman et al., 1987). Plasma NfL levels were about 2-fold higher in patients with inherited peripheral neuropathy CMT than in healthy controls (13.2 vs. 5.2 pg/ml) and correlated with disease severity (Sandelius et al., 2018; Millere et al., 2021). Serum NfL was also significantly elevated in acquired peripheral neuropathy and their levels correlated not only with disease severity and outcome (Mariotto et al., 2018) but also declined with remission (Bischof et al., 2018). These studies suggest that NfL might be a promising biomarker for disease activity monitoring of peripheral neuropathy.

#### Parkinson’s Disease

Plasma NfL levels were about 1.6-fold higher in patients with advanced Hoehn-Yahr stage and patients with PD dementia than in healthy controls (17.6 vs. 10.6 pg/ml) and correlated with disease severity (Lin et al., 2019). Higher baseline plasma levels of NfL were also associated with greater motor and cognitive decline after a follow-up period of 3 years in patients with PD, suggesting value of NfL as a predictive biomarker of disease severity and progression in this disease (Lin et al., 2019; Ma et al., 2021; Zhu et al., 2021). A recent study also suggests that higher serum NfL levels were also associated with dopamine transporter concentration (Ye et al., 2021).

#### Huntington Disease

Plasma NfL levels were about 3-fold higher in patients with HD than in healthy controls (3.63 vs. 2.68 log pg/ml) and also significantly higher in manifest HD than premanifest HD (Byrne et al., 2017, 2018). Increased CSF and plasma NfL appeared in young adult carriers of HD gene mutation approximately 24 years before the clinical onset of symptoms (Scahill et al., 2020). Each CAG (cytosine, adenine and guanine trinucleotide repeat) increase is associated with higher, more steeply rising NfL levels (Byrne et al., 2017).

#### Stroke

Cerebrospinal fluid NfL was first reported to correlate with outcome after aneurysmal subarachnoid hemorrhage (Nylen et al., 2006) followed by the observations of increased CSF pNfH levels in acute ischemic stroke (8-fold at week 3 after stoke, 2.96 vs. 0.35 ng/ml in controls) (Singh et al., 2011). The findings were replicated with the measurements of serum NfL levels that patients with recent subcortical infarcts had higher NfL baseline levels compared to healthy controls (Gattringer et al., 2017; Pujol-Calderon et al., 2019; Peters et al., 2020). Elevated plasma NfL was also associated with poor functional outcome and mortality rate after spontaneous subarachnoid hemorrhage (Hviid et al., 2020a). The elevated NfL levels continued at the 3-month follow-up and seemed to return to normal at 15-month after stroke, indicating that levels of NfL could be a tool for monitoring infarct extent (Tiedt et al., 2018), predicting cognitive function (Peng et al., 2021; Wang J.H. et al., 2021) and mortality in patients with stroke (Gendron et al., 2020). Serum NfL levels also correlate with disease severity, disease progression and 17-year survival in patients with cerebral autosomal dominant arteriopathy with subcortical infarcts and leukoencephalopathy (CADASIL) caused by mutations in the NOTCH3 gene (Gravesteijn et al., 2019). In addition to NfL and pNfH, CSF and serum NfM levels were also elevated in patients with stroke (Martinez-Morillo et al., 2015).

#### Traumatic Brain Injury and Spinal Cord Injury

One month after neurosurgical trauma, there was a distinct peak in CSF (6-fold increase, 2460 vs. 409 ng/ml at baseline) and plasma NfL concentration, which peaked at 1-month post-surgery, returning to baseline after 6 to 9 months (Bergman et al., 2016). Boxers who received severe head impact (>15 hits to the head or experienced grogginess during or after bout) had elevated plasma NfL at 7–10 days after a bout compared to boxers who received mild head impact (<15 head hits) (Shahim et al., 2017). In TBI, both CSF and serum NfL levels were elevated over the first 1–2 weeks compared to healthy controls (Al Nimer et al., 2015; Shahim et al., 2017; Hossain et al., 2019), decreased over 5 years and correlated with measures of functional outcome (Shahim et al., 2020). Similar to TBI, CSF and serum NfL concentrations are also increased in SCI patients compared to healthy controls (Guez et al., 2003), correlated with motor outcome 3–12 months after trauma and minocycline treatment showed decreased NfL levels in a subgroup of injured patients (Kuhle et al., 2015). Care must be taken when TBI patients are over 60 years old or having pre-existing neurological conditions (Iverson et al., 2019). In addition to NfL, serum pNfH was also increased in TBI (Shibahashi et al., 2016) and SCI patients (Hayakawa et al., 2012; Singh et al., 2017) and appears to be a predictive biomarker for outcome.

#### Spinal Muscular Atrophy

Spinal Muscular Atrophy (MSA) is a rare neuromuscular disorder due to a mutation of survival of motor neuron 1 gene that results in the loss of motor neurons and progressive muscle wasting. Baseline levels of CSF NfL (31-fold, 4598 vs. 148 pg/ml) and tau (2.3-fold, 939 vs. 404 pg/ml) were significantly higher in children with SMA than in controls (Olsson et al., 2019). Treatment with nusinersen, a drug that increases the level of SMN protein in the CNS normalized NfL and tau levels which correlated with degree of motor improvement in children with SMA (Olsson et al., 2019). Plasma pNFH levels were also observed to correlate with disease activity and treatment response in infants with MSA treated with nusinersen (Darras et al., 2019).

#### Spinocerebellar Ataxia Type 3

Spinocerebellar ataxia type 3 is a condition characterized by progressive problems with movement due to mutations in the ataxin 3 gene. Plasma NfL levels were about 4-fold higher in patients with SCA3 than in healthy controls (34.8 vs. 8.6 pg/ml) and correlate with disease severity, disease progression and CAG repeat length of ataxin 3 gene mutation (Li et al., 2019; Peng et al., 2020; Wilke et al., 2020). Increased serum NfL appeared in mutation carriers 7.5 years before the clinical onset of symptoms (Wilke et al., 2020).

#### Human Immunodeficiency Virus Infection

Human immunodeficiency virus (HIV) invades brain and leads to the CNS injury, most severely manifesting as HIV-associated dementia with high morbidity and mortality (Price and Brew, 1988). CSF and plasma NfL levels were elevated in HIV infection, especially in HIV-associated dementia (44-fold increase for CSF NfL, 16185 vs. 363 nmol/L in HIV-negative controls), and is markedly reduced after antiretroviral treatment-induced viral suppression (Abdulle et al., 2007; Jessen Krut et al., 2014; Gisslen et al., 2016). Plasma NfL is also negatively associated with neuropsychological performance in HIV-infected individuals and their levels decline with initiation of antiretroviral therapy (Anderson et al., 2018).

#### Prion Diseases

Prion diseases are a family of rare progressive neurodegenerative disorders that affect both humans and animals. CSF and blood NfL levels are significantly higher (about 4-fold increase) in both sporadic and genetic prion disease compared to healthy controls (Steinacker et al., 2016; Thompson et al., 2018; Kanata et al., 2019; Zerr et al., 2021). Increased plasma NfL appeared in adult carriers of prion gene mutation as early as 2 years before the clinical onset of symptoms (Thompson et al., 2021).

#### Hereditary Transthyretin-Mediated Amyloidosis

Hereditary transthyretin-mediated amyloidosis is a condition with adult onset caused by mutation of transthyretin and characterized by extracellular deposition of amyloid and destruction of the somatic and autonomic PNS. Plasma NfL levels in patients with hereditary transthyretin-mediated (hATTR) amyloidosis with polyneuropathy were 4-fold higher than in healthy controls (69.4 vs. 16.3 pg/ml) (Ticau et al., 2019). Levels of NfL at 18 months increased in placebo-treated patients (99.5 pg/ml) and decreased in patients treated with patisiran (48.8 pg/ml), a gene-silencing drug that interferes with the production of an abnormal form of transthyretin (Ticau et al., 2019). The levels of 66 proteins in blood were significant changed following patisiran treatment relative to placebo, with change in NfL being the most significant (Ticau et al., 2019). Moreover, at 18 months, improvement in mNIS + 7 (a robust and clinically meaningful measure of neuropathy progression) compared to baseline in patisiran-treated patients significantly correlated with a reduction of plasma NfL levels.

#### Late Infantile Neuronal Ceroid Lipofuscinosis Type 2

Ceroid lipofuscinosis type 2 (CLN2) disease is an inherited disorder that primarily affects the nervous system. Before treatment in CLN2 patients, plasma NfL levels were 48-fold higher than in healthy controls (153.2 vs. 3.21 pg/ml) and in CLN2 disease, subjects receiving replacement therapy with cerliponase alfa, plasma NfL levels decreased by 50% each year over 3 years of treatment (Ru et al., 2019). Cerliponase alfa-treated patients demonstrated fewer declines in motor and language function than that in historical controls (Schulz et al., 2018). The fold change of CSF NfL compared with healthy controls has been shown varied extensively between individual conditions, with the smallest effect sizes observed in subjective cognitive decline and PD, and the largest effect sizes observed in cardiac arrest, HIV-associated dementia, FTD/ALS, ALS and HD (Rosen et al., 2004; Bridel et al., 2019). The pre-treatment plasma NfL levels observed in CLN2 disease patients is at the high end of neurological disease levels – similar to that seen in ALS, FTD/ALS, HIV-associated dementia and higher than many other neurodegenerative diseases. Even within ALS group, the CSF NfL levels in patients with lower motor neuron signs (346 pg/ml) only had 2.6-fold increase compared with healthy controls (138 pg/ml) while 17.6-fold increase was observed in those with signs of upper motor neuron disease (2435 pg/ml) (Rosengren et al., 1996). Therefore, the fold change in CSF and serum NfL levels could be due to damage to different neuronal compartments in different nervous system regions.

#### Brain Cancer

Neurofilament light chain levels in serum are sensitive to any neuronal damage. As CNS tumors grow bigger and bigger, they could affect function and integrity of neighboring neurons and/or may cause increased intracranial pressure that compromises neuronal function. Accordingly, levels of serum NfL in patients with CNS tumors with progressive disease were 33-fold higher than in healthy controls (239.3 vs. 7.2 pg/ml) and vary closely with tumor activity (Hepner et al., 2019). Similarly, neurons could be damaged by the infiltration of the brain metastasis in the brain parenchyma, brain compression caused by metastasis, vascular disturbance and toxic products diffusing from tumor cells (Zhang and Olsson, 1997). In fact, serum NfL levels in patients with metastatic solid tumors with known brain metastasis were 19-fold higher than in healthy controls (142.3 vs. 7.2 pg/ml) (Hepner et al., 2019). This finding was later confirmed and expanded that an increase in serum NfL could be detected 3 months before brain metastasis diagnosis and a high level of NfL at time of brain metastasis correlated with an inferior survival (Winther-Larsen et al., 2020; Lin et al., 2021). These studies imply serum NFL is a potential clinical biomarker for both CNS tumors and metastatic solid tumors with brain metastasis.

#### Cardiac Arrest

Neurons in the brain can be damaged due to prolonged oxygen and sugar deprivation within 3 min of the heart stopping. CSF NfL levels were first reported to be increased in adult patients with cardiac arrest (52-fold, 11,381 vs. 217 pg/ml in healthy controls) and highly predictive of poor outcome (Rosen et al., 2004). This finding was later confirmed (Rosen et al., 2014) and also with plasma (Wihersaari et al., 2021) or serum NfL levels (Rana et al., 2013; Disanto et al., 2019). Recently, similar findings were also reported in pediatric patients with cardiac arrest (Kirschen et al., 2020). Cardiac arrest over 3 min can lead to not only hypoxic-ischemic brain damage but also reperfusion injury, the restoration of blood flow after resuscitation placing oxidative stress on the brain as pooled toxins flood already-damaged tissues (Sekhon et al., 2017). Future studies in large dedicated cardiac arrest cohorts with serial longitudinal measurements of serum NfL and parallel analyses to assess changes caused by hypoxia, ischemia and reperfusion in brain are warranted.

#### Delirium

Serum NfL levels in delirium in hip fracture patients were 1.7-fold higher than in controls (94 vs. 54 pg/ml) (Halaas et al., 2018) and plasma NfL was associated with delirium severity (Fong et al., 2020) independent of changes in inflammation (Casey et al., 2019). In addition to elevated NfL, higher serum pNfH levels also correlated with more severe postoperative delirium (Inoue et al., 2017; Mietani et al., 2019). These results suggests NfPs can be sensitive markers of neuronal injury associated with delirium.

#### The Value of Neurofilament Proteins in Differential Diagnosis Is Limited

Although NfPs are not disease-specific, they may have limited utility in differential diagnosis in some cases. Some neurodegenerative diseases share part of their symptomatology and neuropathology, making it difficult to differentiate between them. The differentiation between multiple system atrophy (MSA) and PD is difficult, particularly in early disease stages. Increased CSF NfL may offer clinically relevant, high accuracy discrimination between MSA and PD (Herbert et al., 2015) and also between PD and other atypical parkinsonian disorders including progressive supranuclear palsy and corticobasal degeneration (Constantinescu et al., 2010; Ashton et al., 2021a). The overlap of FTD and ALS has been well documented in FTD patients with co-morbid motor neuron degeneration and in ALS patients with frontotemporal dysfunction (Lomen-Hoerth, 2011). CSF NfL levels are higher in ALS than in FTD (Skillback et al., 2017) and also significantly higher in patients with FTD-ALS than in patients with FTD without ALS (Pijnenburg et al., 2015). CSF pNfH has also been shown to be a better biomarker than CSF NfL in differentiating ALS from other diseases mimicking ALS symptomatology (Poesen et al., 2017). Early symptoms of patients with FTD typically do not include memory impairment but instead often manifest changes in their behavior, personality and social interaction, which are often confused with symptoms occurring in psychiatric disorders. About 50% behavioral variant FTD patients received a prior diagnosis of a psychiatric disorder in a large retrospective study (Woolley et al., 2011). Patients with FTD have significantly higher serum NfL levels than patients with psychiatric disorders (Al Shweiki et al., 2019; Katisko et al., 2020), suggesting NfL as a promising tool to help differentially diagnose FTD and psychiatric disorders.

## Current Research Gaps and Potential Development of Neurofilaments as Biomarkers

### Blood-Brain and Blood-Cerebrospinal Fluid Barriers

The effects of blood-brain barrier (BBB) and blood-CSF barrier (BCB) on serum NfP levels are not fully understood. Aging and neurodegenerative disease can cause increased disruption of the BBB (Sweeney et al., 2018) which could contribute to the elevated levels of serum NfP signals observed in these conditions. Recent evidence suggests that serum NfL level does not correlate with opening of the blood brain barrier after cranial irradiation (Kalm et al., 2017). Consistent with this finding, higher CSF/serum-albumin ratios were observed in FTD-3 patients, but this did not affect the significant associations among serum NfL levels and pre-symptomatic, symptomatic CHMP2B (charged multivesicular body protein 2B) mutation carriers and healthy family controls (Toft et al., 2021).

### The Exact Form of Extracellular Neurofilament Proteins and Degradation Fragments

Because full length NfL proteins have never been detected in CSF and blood, it seems likely that most or all of the NfL detected in the CSF or serum are peptides generated from partial degradation of NfL in neurons or after their release. The identity and form(s) of NfPs detected by the commonly used NfL antibodies is not fully clarified. Recent studies suggest that a 22 kDa degradation fragment could be the detected plasma signal of NfL since it is also increased in ALS patients (Lombardi et al., 2020). The peptide species of INA and PRPH in CSF and plasma are not known. If a Nf subunit such as INA is fully and rapidly degraded into amino acids upon release from neuronal compartments into blood, then no signals of Simoa assay can be measured and no value of utility as a biomarker. Determination of the form of detected NfP immunosignals (full length or degradation fragments) in blood will not only impact their utility as blood biomarkers but also help to better understanding the pathophysiological process in a given neurological diseases.

### The Relationship Among Different Neurofilament Subunits

Neurofilament proteins are not identical and each has a distinct structure and could potentially have differential diagnostic value as biomarkers. The relationships among NfPs are complex and interrelated. When NfL is absent in mice, NfH levels decline most, followed by the decreased levels of NfM and PRPH (Yuan et al., 2012) and the levels of INA is only marginally declined (Yuan et al., 2003). When NfM is absent in mice, NfL levels decline most, followed by the lowered levels of NfH and INA (Yuan et al., 2006). NfL and NfM are co-regulated in mammalian brain and only marginally affected by the deletion of NfH, INA or both (Yuan et al., 2006). The first ELISA for NfPs was developed (Rosengren et al., 1996) prior to the recognition of INA and PRPH as additional Nf subunits (Yuan et al., 2006, Yuan et al., 2012). NfL is the most intensively studied subunit as a biomarker followed by phosphorylated NfH, especially after introduction of a highly sensitive digital assay (Gisslen et al., 2016).

Despite less attention being paid to NfM, INA and PRPH as biomarkers in neurological diseases, their potential utility is considerable. In addition to the well-established increase of NfL during aging, a highly significant increase in the levels in CSF of both phosphorylated and non-phosphorylated NfM and NfH are also seen in aged individuals as compared with young controls (Hu et al., 2002). NfPs are an integral part of neurofibrillary tangles in AD brain (Rudrabhatla et al., 2011) and C-terminal phosphorylation sites of both NfM and NfH are 4- to 8-fold more abundant in AD compared with control brain (Rudrabhatla et al., 2010). Levels of specific phosphorylation sites on NfM and NfH in blood could potentially be used as a biomarker to discriminate AD from normal brain aging and other neurological conditions.

Alpha-internexin is enriched in CNS and its prominent aggregation in Nf inclusion disease (Cairns et al., 2004) could qualify INA as a CNS-selective biomarker. However, the intact form of INA is difficult to detect in laboratory practice due to its instability. A possible solution could be to test for blood levels of its more stable degradation products. INA was identified by proteomics as a novel biomarker in the CSF of patients with hemorrhagic stroke (Martinez-Morillo et al., 2014). In contrast to INA, PRPH is enriched in PNS (Yuan et al., 2012) and therefore could potentially be developed as a PNS-specific biomarker. Moreover, PRPH is also sensitive to diffuse axonal injury (Liang et al., 2019) and its aggregate-inducing isoform Per 28 is upregulated in ALS and is associated with disease pathology (Xiao et al., 2008). A recent report suggests high serum levels of PRPH might be a general biomarker of axon disorders of lower motor neurons (Sabbatini et al., 2021). Future studies should therefore aim to develop assays of appropriate specificity for each of the NfP subunits or degradation fragments to explore the complementary information they may contribute to NfP pathobiology and use as biomarkers.

### Stable Isotope Labeling Kinetics Coupled With Mass Spectrometry

The levels of NfP and peptide in CSF and blood depend on the rates of synthesis of NfPs or mechanism and rates of NfP peptide release. A recently developed stable isotope labeling method coupled with mass spectrometry may be useful to define the kinetics of NfP turnover in healthy individuals, with aging and in patients with neurological conditions associated with elevated NfP signals in CSF and blood. Special attention should be paid to the extremely slow turnover of NfPs incorporated into the filamentous lattice in axons (Nixon and Logvinenko, 1986; Yuan et al., 2015a). This method uses hours-long infusions of ^13^C and ^15^N stable isotopes before measuring the labeled proteins in CSF, blood or brain tissue samples (Bateman et al., 2006; Paterson et al., 2019). The incorporation of newly synthesized labeled proteins gradually increases until a steady state is reached. Following stop of infusions, the proportion of the labeled amino acid in the target protein gradually declines as a result of protein clearance or degradation. Alterations in the isotopic enrichment of the target proteins allow the calculation of protein synthesis and clearance rates from the ratio of labeled to non-labeled protein. This method was used to measure the kinetics of tau isoforms and fragments in human CNS (Sato et al., 2018). The elevated CNS tau levels in AD patients was initially interpreted as resulting from passive release of this protein by degenerative neurons. However, results from stable isotope labeling kinetics (SILK) studies suggest that the bulk of tau in human CSF is released by an active process that is stimulated by neuronal exposure to aggregated amyloid-beta. On the one hand, the concentration of NfPs in CSF or serum measured at a given time represents a static biomarker whose equilibrium could be affected by various factors. On the other hand, NfP-SILK can provide dynamic measure of production and clearance of newly synthesized NfPs that might provide a more detailed understanding of the mechanisms underlying these alterations in NfP levels.

### Confounding Factors

Since there are significant variations of measured blood NfL levels among different methods and labs, standardization of blood NfL measurement globally is needed. Care must be taken when interpreting results obtained in different studies. Community-based large populations of healthy individuals are required to generate normative data for reference intervals. As discussed earlier, there are numerous demographic, life style, and comorbidity factors that potentially influence NfP levels in biological samples. With the increasing use of blood assays, variables such as exercise (Joisten et al., 2021), blood volume, body mass index need to be considered (Manouchehrinia et al., 2020; Perino et al., 2021). Trace amounts of NfPs relative to those in neurons have been reported in erythrocytes (Granger and Lazarides, 1983; Terasawa et al., 2006), T lymphocytes (Murphy et al., 1993), podocytes (Wang et al., 2018), and oocytes (Takahashi and Ishizuka, 2012), which could be confounds in certain disease conditions. Because blood NfL alteration is associated with aging, future studies are also needed to establish the age-adjusted normal values of serum NfL levels across all age groups. The recent establishment of reference intervals of serum NfL in 342 Scandinavian reference subjects from 18 to 87 years of age is a step in the right direction (Hviid et al., 2020b). Comparative studies of two or more neurological disorders will be valuable to clarify the relative magnitude of change and its disease significance using the same methodologies. Sporadic AD patients are often older individuals associated with higher prevalence of cardiovascular conditions that is also associated with CNS ischemic damage and subsequent release of NfPs into blood (Gattringer et al., 2017). Co-existing peripheral neuropathy with CNS diseases may also weaken the correlation between CSF and serum NfP signals. Longitudinal measurements should also be encouraged to minimize intra- and inter-individual variation due to transient confounding variables and emerging co-morbidities (Khalil et al., 2020; Liu S. et al., 2020).

## Conclusion

The development of minimally invasive ultrasensitive assays of NfPs released from neurons into in blood has increased the potential use of NfPs as biomarkers especially for repeated measurements during longitudinal studies such as in MS. The degree of elevation of NfPs in serum could easily differentiate behavioral FTD from primary psychiatric disorders where significant clinical overlaps of these two conditions exist and the sensitivity and specificity of structural and functional imaging methods remain imperfect. Monitoring the kinetics of NfPs in blood can increase our ability to assess disease activity, neuronal injury, and neurodegeneration in real time and to measure treatment effectiveness. Much interest has been focused on the detection of blood NfPs by high-sensitivity assays as a surrogate marker of neuronal structural damage and degeneration. However, the majority of these reports are cross-sectional, more longitudinal data are required to better elucidate the place of NfPs in the clinical settings. Due to their lack of specificity for a given disease, NfPs will most likely be of limited value as a diagnostic tool except when levels drastically differ between two conditions with similar clinical presentations. No single test or value of NfPs can currently be used to rule in or exclude the diagnosis of a specific disease. Nevertheless, NfPs can potentially be used to monitor disease progression and the effects of therapeutic intervention in combination with clinical judgment in almost any neuronal injury and neurological diseases. Serum NfPs are relatively easily measured. Treatment-induced decrease in blood NfPs levels as a complement to the more lengthy process of measuring clinical outcomes may, in the future, be more important in the validation and regulatory approval of new drugs for neurological conditions.

## Author Contributions

AY and RN wrote the manuscript and have approved it for publication. Both authors contributed to the article and approved the submitted version.

## Conflict of Interest

The authors declare that the research was conducted in the absence of any commercial or financial relationships that could be construed as a potential conflict of interest.

## Publisher’s Note

All claims expressed in this article are solely those of the authors and do not necessarily represent those of their affiliated organizations, or those of the publisher, the editors and the reviewers. Any product that may be evaluated in this article, or claim that may be made by its manufacturer, is not guaranteed or endorsed by the publisher.
